# The nebula of chronicity: dealing with metastatic breast cancer in the UK

**DOI:** 10.1080/13648470.2022.2041547

**Published:** 2022-03-11

**Authors:** Cinzia Greco

**Affiliations:** Centre for the History of Science, Technology and Medicine (CHSTM), University of Manchester, Manchester, UK

**Keywords:** Metastatic breast cancer, chronic disease, United Kingdom, medical professionals, patients’ experiences

## Abstract

In this article, I explore how the concept of chronicity is mobilised by different actors in reference to metastatic breast cancer (MBC) and the transformation of the condition as a consequence of medical innovations. I do so by using data collected in the UK between 2017 and 2019 through in-depth interviews with medical professionals involved in the treatment of MBC and with patients living with MBC. I show how chronicity appears as a multidimensional and uncertain concept, which I analyse through the image of the nebula. While the medical literature tends to consider MBC chronic or on route to chronicisation, the medical professionals interviewed were uncertain as to whether MBC can be considered a chronic disease, and attempted to discuss chronicity through survival times, the kind of management possible for the disease, and how it compares to other conditions more commonly considered chronic. In some cases, the patients considered the idea of chronicity a source of hope or a way to link their condition to those of people with other diseases; however, they generally rejected the definition as inappropriate for their experience of the illness. Analysing the fluid uses of the concept of chronicity in the case of MBC contributes to the debate within medical anthropology on how medical categories acquire different values and uses and on the circulation of meanings between the biomedical context and the patient experience.

In this article, I explore recent evolutions in the treatment of metastatic breast cancer (MBC). Triangulating data derived from interviews with medical professionals and patients, and an analysis of medical literature, I examine whether MBC can be defined as a chronic disease. Investigating the shifting status of MBC allows me to disentangle the complexities and uncertainties within the definition of chronicity and ultimately argue that the concept is unstable and heterogeneous yet can be understood through comparison to a nebula.

There are two reasons to examine chronicity in MBC. First, despite routine use, the concept of chronicity has historically lacked a precise definition (Weisz 2014). Second, for MBC in particular, chronicisation claims can be linked to previous studies of economies of hope in oncology (Delvecchio Good et al. 1990; Therond et al. 2020) that have considered how new treatments are charged with hopes regarding their efficacy. In the case of breast cancer there is a pervasive optimist rhetoric, expressed by the ‘pink’ narrative, that over-emphasises both the possibility of ‘defeating’ breast cancer and the role of early diagnosis. This rhetoric has rendered MBC, the stage linked to death, mostly invisible (Davis 2016; Jacobson 2018). In this context, presenting MBC as chronicised – and, therefore, under control – denotes a different way of inscribing it within the optimistic rhetoric of breast cancer. Comparing medical literature with the experiences of both medical professionals and patients shows the different ways the uncertain concept of chronicity is defined in the three contexts. Furthermore, it indicates how defining MBC as chronic enables medical literature to present innovation in breast cancer positively, without however convincing either all medical professionals or (and even less so) patients.

## The blurred boundaries of the concept of chronicity

The historian of medicine George Weisz argues that chronicity is an ‘imprecise and elastic’ concept (2014, 7) adopted in contemporary biomedicine as a result of its development within US healthcare policies. In the first half of the twentieth century, public authorities gradually shifted their attention from infectious diseases to manageable long-term conditions; simultaneously, there emerged a greater consideration for the well-being of the older population. ‘Chronic’ can be used to describe different situations and can be difficult to clearly define. While length is the central dimension of chronic conditions, chronicity is also increasingly described multidimensionally, even in biomedical contexts. For example, Walker (2001) observed how, along with length, chronic conditions can be described through biological markers or impact on lifestyle. One of the most extensive analyses within medicine is that of O’Halloran, Miller, and Britt (2004), who identify other potential dimensions, including gradual onset, recurrent or deteriorating development, poor prognosis and significant sequelae.

Some of the most influential works on chronicity – within both sociology and nursing (Bury 1982; Charmaz 1983; Corbin and Strauss 1984, 1985) – have shown great interest in identifying different typologies of trajectories in the evolution of chronic conditions, but less interest in defining the concept itself. While the trajectory approach has been criticised for some time – because many chronic conditions do not present foreseeable evolutions and do not tend towards normalisation (e.g., Paterson 2000) – there have been attempts to identify regular trajectories also within MBC (Kenne Sarenmalm 2009; Reed and Corner 2015).

Medical anthropologists have researched extensively long-term medical conditions, generating fundamental knowledge about such illness experiences (cf. Whyte 2012). They have shown how chronic conditions are inscribed in specific political contexts that shape the development of illness, rather than being chronic merely because of the ‘natural course of disease’ (Smith-Morris 2010: 21). Although medical anthropology has focused on concept of chronicity itself less than sociology and nursing, it has provided a broader critique of the concept. It has emphasised that a disease’s chronicisation depends on an access to treatments that is not guaranteed globally, and pointed out the limitations of defining chronic conditions in opposition to non-chronic ones, especially contagious or acute diseases. Both lay and biomedical ideas of chronic disease are built around pathologies such as diabetes and hypertension, which are considered chronic because they can be kept under control for a long time and patients often have a good quality of life. However, absent or insufficient access to healthcare can turn otherwise manageable conditions in fatal ones, altering their perception as chronic. Several studies have observed how in countries in the Global South delayed cancer diagnoses and difficulty accessing newer and more efficient therapies can make it extremely difficult for staff and patients to see cancer as a chronic condition (see Mulemi 2008 for Kenya; Livingston 2012 for Botswana; and Nédélec 2018 for the Ivory Coast). Thus, the study of chronic conditions considers their social context using a syndemic approach (Weaver and Mendenhall 2014), which has shown how chronic conditions, such as diabetes, can interact with health problems, such as depression, and how the interaction between two or more conditions is influenced by other factors, such as gender or migration status.

This approach is consistent with the attention, within the field of medical anthropology, to deconstruct the boundaries of chronicity and the efforts to re-examine these biomedical categories and dichotomies. For example, Manderson and Smith-Morris (2010, 3) argue that the chronic/acute dichotomy ‘inaccurately captures the lived experience of illness over time and in different settings, while minimizing the social and cultural contexts and globalizing forces that pattern health and well-being’. Additionally, chronic conditions have been commonly described as non-communicable diseases, while acute conditions are often considered to be also communicable. These categorisations do not consider the contextual nature of the experience of illness. For example, the introduction of antiretroviral therapies has made HIV/AIDS a disease that many now define as chronic (cf. Whyte 2012), while ‘treatment as prevention’ and pre-exposure prophylaxis are changing the disease’s communicable nature (Genre and Panese in this issue).

Furthermore, it is difficult to know where to draw a line between chronic and terminal. Baszanger’s (2012) literature analysis suggests that the idea of chronicity emerged for advanced cancer as a consequence of the introduction of multiple lines of treatment and the blurring of the concepts of curative and palliative treatment that were more distinct until the 1970s. Lerum et al. (2015) in their study of Motor Neurone Disease (MND) in Norway, show how medical professionals working on MND recognise the importance of the categories of chronic and terminal and use them routinely to guide their work. However, both medical professionals and informal carers have difficulties in drawing a distinction between chronic and terminal. Such difficulty is mostly a result of substantial uncertainty regarding potential survival times, but is also linked to the ways categorisation influences disease management and life-or-death choices. For example, a patient classified as having terminal MND is allocated more primary care resources than a patient with chronic MND; additionally, while terminal conditions do not entail an obligation to attempt resuscitation, chronic conditions do. Lerum and colleagues link the blurring between terminal and chronic specifically to technological advances that have lengthened survival times with MND through highly invasive support systems.

Chronicity is far from the only uncertainly defined medical concept; in many cases, definitions of medical conditions themselves can be highly contested, as in the case of ADHD (Rafalovich 2005; Nielsen 2019). However, conditions such as ADHD are at the centre of extensive debates on their adequacy, whereas chronicity is used routinely and unproblematically, but without a real shared definition. The complex and variable conceptualisation of chronicity can be understood by comparing it with the nebula. In astrophysics, a nebula is a cloud of gases and dust that can be either the by-product of a star explosion or a cradle of new stars. Nonetheless, a nebula’s materials remain subject to gravitational forces that can give the compound a more defined structure. Similarly, the concept of chronicity is not just unclear or lacking a shared definition. Instead, in the extant definitions for chronicity we can find different structuring principles that can push the concept in different directions. For the medical professionals I interviewed, these structuring principles are the dimensions of chronicity previously discussed, which they use to try to make sense of MBC. However, for the patients, while there are hopes linked to longer survival, it is difficult to understand where they are within the nebula or where they are going (for more details on this uncertainty, see Greco 2022). Considering the concept’s instability, it is worth questioning why chronicity attracts so much attention and why it is so largely used. Part of its success, both in medical and lay contexts, lies in the vagueness of the term, and in the possibility of evoking different situations without requiring a precise definition. In biomedicine, chronicity is often associated with a medical success. In the case of cancer, the idea that it can become a chronic condition contributes in transforming its perception from death sentence to manageable condition, with its framing as chronic impacting how public authorities, medical professionals and patients deal with it. In the case of MBC in particular, the analysis I present here of the fluid uses of the concept of chronicity contributes to the debate within medical anthropology surrounding how medical categories acquire different values and uses, and the circulation of meanings between the biomedical context and the experiences of the patients.

## Methodology

The data presented here derive from research I conducted in the UK between 2017 and 2018 interrogating the relationship between MBC and chronicity and the degree to which MBC could be considered a chronic disease. The concept of chronicity was a prism through which I aimed to ‘refract’ the different layers of the MBC experience. The research was guided by the extended case method approach (Burawoy 1991), which links ethnographic data to dimensions of analysis at a larger level than the one observed, and in which the data are compared with the theories existing in literature, in order to identify where the research results deviate from already formulated theories. I explored the degree to which definitions of MBC as chronic, as presented in medical literature and debated among patients’ associations, was pertinent at an ethnographic level.

In the analysis I further followed Fainzang’s (1996) invitation for medical anthropology ‘to distance itself from some of the ideas elaborated by other disciplines’ (473). My analysis of the medical literature is not a simple literature review, but a critical examination of the claims of chronicity. Similarly, I use the interviews with medical professionals to understand their point of view, but also to highlight the differences between their views and those expressed in the literature, and locate the different definitions within cancer’s larger social context to offer a social interpretation of the rationales of the different uses of the concept of chronicity.

This article is based on in-depth interviews with two groups. I conducted 16 interviews with medical professionals (10 oncologists, 4 nurses, 1 radiotherapist and 1 biologist) with experience in treating MBC. I further conducted 10 interviews with patients with an MBC diagnosis. The interviewees were contacted through medical institutions and, for what concerns the patients, through patients’ associations. The interviews with medical professionals lasted between 30 and 50 minutes and focused on their professional experience with MBC, their vision of therapeutic innovations and their vision of the relationship between MBC and chronicity. One of the aims of the interviews with medical professionals was to elicit the kind of clinical knowledge which is not included in medical literature because of a restrictive understanding of what constitutes evidence-based medicine (cf. Greenhalgh, Howick and Maskrey 2014). The interviews with patients were longer, lasting between 30 minutes and 3 hours. In these interviews, I explored their life and health before their first breast cancer diagnosis, their illness experience following diagnosis (and, for patients who had not been diagnosed with MBC directly, the difference between the early stage and the metastatic experience), their relationship with medical professionals and the impact of the illness on their personal, social and working life, including the help received from relatives and friends. In the interviews with the patients I did not ask explicitly whether they considered MBC a chronic condition because this definition can be perceived as dismissive by some patients; nevertheless, several women spontaneously discussed chronicity and MBC, confirming the importance of the category also among the patients. The patients interviewed were diverse in terms of age (mid-30s to late-70s) and years since the metastatic diagnosis (ranging from a few months to 10 years), but minorities and women from lower social classes were scarcely represented. Given processes of self-selection, the interviewees probably also had better health conditions and more involvement in activism than the average among patients with MBC in the UK. Results from an ongoing study that began in 2019 on breast and lung cancer innovation and policies in the UK, for which I have also interviewed medical professionals and patients, provide the present analysis with further insight on how MBC finds place in the larger organization of breast cancer treatment in the UK. All the names used here are pseudonyms, and I have altered some minor details in order to safeguard the interviewees’ privacy.

I integrated the data derived from in-depth interviews with observations of medical conferences and a systematic literature review for the period 1979–2016. The literature review was conducted using PubMed and, to allow full-text search, Google Scholar. The resulting corpus of publications consisted mostly of medical articles, with some medical books, and publications from allied disciplines, including social studies of health. The literature review acted as a baseline against which I compared in particular the interviews with medical professionals. This allowed me to elicit the differences between the literature and the clinical experience in terms of how chronicity is defined and applied to MBC. Analysing the three sources of data together allowed identification of the multiple and contradictory dimensions of chronicity in the case of MBC, and of the ways in which the concept, fluid and irregular like a nebula, is gradually increasing its influence on the perception and experience of MBC.

## Metastatic breast cancer in public debate and medical literature

Breast cancer is one of the cancers for which available treatments allow long-term (more than 5 years) survival for most patients (cf. Timmermann 2013). In most cases, patients with early stage breast cancer (ESBC) can live longer than 5 years, and 70% of patients can live for 20 or more years, with some variation according to age at diagnosis. The social attention concerning breast cancer focuses on early detection and treatment (cf. Löwy 2009). However, even after the end of treatments, life post-ESBC is characterised by long-term side effects and by the continued possibility of relapse (for a larger discussion of the impact of possible relapse in cancer see e.g. Skowronski et al. 2019). A significant number of women diagnosed with ESBC will develop MBC, which is the stage of the disease that is linked to mortality. Depending on the initial stage and the biology of the tumour, it is estimated that 20 to 70% of ESBC patients will develop metastasis (Dewis and Gribbin 2009).

Epidemiological studies have shown increased median survival for MBC beginning in the 1990s, attributing this phenomenon to the new drugs that were introduced in that period (e.g., Andre et al. 2004). Such drugs include aromatase inhibitors, chemotherapeutic drugs like taxanes, and, more recently, monoclonal antibody therapies. These innovations have increased the capacity to treat specific cancer profiles – oestrogen receptor-positive, progesterone receptor-positive and HER2-positive cancers. Meanwhile, for ‘triple-negative’ tumours, specific treatments are still lacking, limiting median survival time. The average survival time for patients with MBC is around three years (Gobbini et al. 2018). However, many patients survive 5 years or more, especially those with bone lesions or fewer lesions.

Some cancer associations, such as Europa Donna, seem to embrace the idea that progress in the treatment of MBC is transforming the condition into a chronic one (Europa Donna n.d.), while other associations are more critical. In 2008, the late Barbara Brenner, executive director of Breast Cancer Action until 2010, alerted about the dangers of defining MBC as chronic: ‘Using the term chronic implies that breast cancer is a manageable disease, and downplays the reality that it is far too often fatal. It also diminishes the fact that we are in desperate need of better treatments (Brenner 2008)’. As we will see in this article, several patients interviewed agreed with Brenner, rejecting the definition of MBC as chronic because of the association of chronicity with conditions such as diabetes and hypertension, conditions perceived as non-life-threatening, and sometimes even non-life-altering. However, chronic seems to be an elastic category undergoing important transformation, precisely because new pathologies are now included under its broad umbrella.

My analysis of the medical literature on MBC and chronicity shows how, similarly to the public debates, the term ‘chronic’ is often used to describe MBC also in medical publications, but the definition of chronicity remains rather opaque.

The medical literature began presenting MBC as a chronic condition as early as 1979 (Wilson 1979) and my analysis of the literature published up to 2016, conducted using PubMed and Google Scholar, shows that 268 publications described MBC as a chronic condition and only 7 explicitly opposed the definition. Most publications based their definition of chronicity on standardised survival measures, such as median survival time (e.g., Andre et al. 2004) or survival rate five years after diagnosis (e.g., Chen, Parmar, and Gartshore 2014). With the discovery of biomarkers that act as targets for new treatments, some publications began to limit their definition of chronicity to specific subtypes of MBC, such as HER2+ (e.g., Soleja and Rimawi 2016) or ER+ (e.g., Redfern et al. 2016). For some of the literature, chronicity was a promise for the near future. That is, when discussing specific medical innovations or encouraging lines of research, results are often presented as developments soon to chronicise the condition; in some cases, chronicisation referred to longer survival times, in others no definition of chronicity was given. However, taken together, the promises of the literature do not seem to have always been fulfilled. For example, while the first publication mentioning chronicity in relation to MBC envisaged the condition in general soon becoming chronic in the near future in 1979 (Wilson 1979), as recently as 2016 there are other publications that have considered only HER2+ MBC likely to become chronic in the near future (Soleja and Rimawi 2016). This uncertainty mirrors the nebulous and vague character of the concept of chronicity.

Although the medical literature surrounding MBC rarely discusses dimensions of chronicity other than the condition’s duration, in some cases the fact that the condition is considered chronic is presented as a reason to give more attention to the quality of life of patients (e.g., Orlando et al. 2007) or further developing supportive care (e.g. Lam et al. 2014).

## ‘We cannot be complacent’: Medical professionals and chronicity

As discussed, the median survival time for MBC has increased in the past thirty years. Among the medical professionals interviewed, those with several years of experience were generally more open to considering MBC as chronic. For instance, according to Dr Brad, ‘It’s more true for some patients than for others, because of the differences in prognosis. With that aim of kinda keeping people well, that’s what you do with chronic conditions.’

Meanwhile, Dr Thomas, while sceptical in general, recognised that ‘more and more [MBC] is being considered a chronic disease, because we have so many more treatments for it now, and people are surviving much longer nowadays than they did back in the 1970s’. Dr Maria was one of those most convinced that MBC could be defined as a chronic disease, but she added that ‘however patients don’t like that. [There is a] research group of patients here who advise us […] and they think it’s undermining the severity of the condition, ‘cause you will call diabetes a chronic condition, but the perceived threat is not the same.’

As pointed out in the previous section, MBC is segmented into subtypes with different availability of treatments. The medical professionals I interviewed frequently mentioned that certain subtypes have a better prognosis, and that the evolution of the disease for those subtypes could be considered as chronic. For example, according to Dr Noah, ‘you can subdivide breast cancer into actually a number of different conditions, based on the characteristics of the cancer, the molecular phenotype of the cancer, how the cancer is metastasising’. Other medical professionals, such as Dr Maria, agreed with this notion: ‘certainly hormone receptor-positive breast cancer can easily be called a chronic disease; the HER2 positive which responds to treatment probably falls into that group’.

However, even subdividing MBC into distinct biological conditions left open the question of defining what chronicity is. One interviewee, Dr Trevor, showed his perplexity about the definition of chronicity:

I don’t really know what the definition of chronic disease is, I suppose a chronic disease would be a disease that you live with… that can be managed and that doesn’t lead to deterioration in your quality of life, and doesn’t kill you. But the timescale for that chronicity is… I don’t know…

This excerpt indicates doubts about the concept of chronicity itself. Here, idea of the nebula is useful to explain how some interviewees approached the problem. Given the irregular and blurred borders of the concept of chronicity, some interviewees attempted indirect approaches to define it. They tried to identify an order in the nebula examining different gravitational forces – different dimensions of chronicity – in order to understand what a chronic condition implies and whether it is applicable to MBC. This included comparing MBC with other diseases for which there is more agreement regarding chronicity, or discussing what kind of management is possible for MBC and whether this makes it chronic. Nonetheless, there were still different opinions among the interviewees on whether MBC is chronic. Some emphasised that progress in the treatment of MBC cannot be compared to progress in the treatment of diabetes or HIV/AIDS, while others argued that conditions usually considered less threatening than cancer, such as heart failure, have survival times similar to those of MBC. For example, while sceptical that MBC could already be considered chronic, Dr Harry said

Metastatic breast cancer is still much more serious than a diagnosis of diabetes. But you could compare it to, say, a diagnosis of heart failure. Heart failure it’s not something that they show survival trends for, but half the people with heart failure die within five to ten years, so [it] cannot be cured. So it depends which disease you are comparing it to.

Several medical professionals found that neither the acute nor the chronic label adequately describe MBC, with Dr Luke proposing an alternative: ‘another definition might be a series of acute episodes linked together by areas of more chronic stabilisation’. This definition seems to support Barbara Brenner’s position mentioned above, which considered MBC as a ‘*recurrent*, not chronic’ condition. This definition also has the advantage of not erasing the difficulties of managing the condition, as recognised by references to acute episodes. Dr Maria also used the kind of management possible as a criterion for discussing the chronicity of MBC:

If you look at the perspective for services, typically the chronic conditions are well-managed by the general practitioner [such as] diabetes, high blood pressure. Heart failure [also] more or less is managed by general practitioners. With cancer, people continue with treatment [and] we still see them in the hospital.

The fact that patients continue to need specialist care is also the reason why there has not been a significant movement towards self-management in MBC as there has been in conditions such as diabetes. On a similar topic, Isabella, a nurse and cancer services manager, describing the impact of new therapies for MBC on cancer services, said that:

there are so many more lines of treatment [for MBC] now and our oncologic clinic has been pretty much at the same capacity for the last 13 years, all the new treatments are having a massive impact on that, so we are trying to respond by saying: ‘OK which patient do we really not need to see any more?’

Continuing, Isabella suggested that to make room for the needs and the difficulties associated with side-effects of the new lines of treatments for MBC, certain services might decide to reduce the follow-ups for patients diagnosed with ESBC. The combination of these two elements – the reduction of the follow up for ESBC patients and the increase of the number of lines of treatment available for MBC patients – reinforces the notion that ESBC is curable while MBC is manageable and, in some case, even a chronic condition.

The interviews sketch a more complex picture of what a chronic condition is, suggesting a multidimensional conception of chronicity. However, the dimensions used changed from interviewee to interviewee, and the definitions were often tentative, showing that chronicity is not only multidimensional but has different aspects that are invoked in different cases. Comparisons with other diseases that are more commonly described as chronic (including diabetes, hypertension and HIV/AIDS) and the ways in which the disease is managed represent two such dimensions that the medical professionals have employed in the interviews. For example, according to many medical professionals, the need to be treated in specialised cancer centres, rather than by GPs, is one of the reasons why it is still not possible to consider MBC truly chronic; thus, it remains, as Dr Maria reiterated, ‘not quite a typical chronic condition’.

Even when it was likened to a chronic disease, MBC was not presented as a typical chronic condition. Instead, this definition was considered applicable only to certain subtypes. For example, some interviewees explicitly identified triple-negative MBC as a condition that could not be considered chronic, with none of the interviewees including it in the definition of chronicity. This diversity within MBC, together with the difficulty, common to every patient, of foreseeing the disease’s evolution, contribute to create a nebulous understanding of MBC as a condition that can be considered chronic, but only given certain conditions. Furthermore, while some medical professionals were willing to recognise a partial and atypical chronicity in MBC, others considered such a description to be inappropriate. For example, according to Lisa, a breast cancer nurse, ‘my concern with metastatic disease is [that] if we talk about it being a chronic illness, that you set some people’s hopes too high’. Dr Mark, an oncologist who has worked on breast cancer treatment for more than thirty years, was also convinced that MBC was not a chronic condition, although he saw a purpose for such a definition:

The emphasis on chronicity hopefully means that for much of the time the patient has metastatic breast cancer we aim to treat them as somebody who is living with breast cancer rather than dying of breast cancer. So, the chronic element I think is relevant because it acknowledges that they have got a life to get on with while they have got the metastatic breast cancer, but I still do not think we can be complacent in thinking that we turned it into a truly chronic condition.

In this interview extract, chronicity is clearly presented as useful insofar as it can help refocus the therapeutic relationship onto the patients and their quality of life. However, the reference to ‘complacency’ highlights the problems still present for MBC, and shows how part of the medical professionals disagree with the presentation of MBC as an already chronic condition in much of the medical literature.

## ‘Some people would call it a chronic illness’: patients and MBC

As discussed in the introduction, the invisibilisation of MBC in the ‘pink’ rhetoric about breast cancer is one of the problems linked to the condition. Many of the patients I interviewed felt they were ‘invisible’ and ‘written off’, as Abby, a patient in her fifties, put it during her interview. Nancy, a patient in her forties, emphasised how she had felt left out since her diagnosis: ‘I’m not less important because I’m incurable’.

Some of the women I interviewed were prepared for the possibility of the cancer returning, even several years after the primary diagnosis, others had not been informed of this risk. For these patients, the metastatic diagnosis was a particularly traumatic moment, with the balance they might have started to find after their first diagnosis shattered by the uncertainty that accompanies an MBC diagnosis. For example, Nancy emphasised how unexpected her diagnosis was, with no medical professional having ever mentioned the risk of relapse: ‘[It is important to tell] people what secondary cancer is, what metastatic cancer is, because people often think that secondary is cancer for the second time, not a primary cancer that has spread’. Nancy considered the term ‘secondary breast cancer’ to be misleading, seemingly referring to a local relapse – a *second* cancer in the breast – rather than to the stage of the disease at which cancer has spread outside the breast to involve other organs. In contrast, the term ‘metastatic’ does not feature this ambiguity. Given that, in most cases, ESBC is treated to reach the status of ‘no evidence of disease’, and many patients never experience metastasis in their lifetime, it is more difficult for medical professionals to discuss the possibility that cancer might come back and that this can happen even several decades after the first diagnosis (Pan et al. 2017).

At the same time, a new definition of MBC as ‘treatable not curable’ condition has begun to be used. Following the initial shock of the diagnosis, patients with MBC start a sequence of treatments that is somewhat similar to that undertaken for ESBC – potentially including some or all of chemotherapy, radiotherapy and immunotherapy – but this time with a different aim. Daisy, a patient in her sixties, perfectly captured this situation:

The first time [when I was diagnosed with early breast cancer] I was told that there was a good chance that it was treatable, obviously this [MBC] is manageable but not curable, so it is a terminal diagnosis. So that’s very, very different. I think that the first time I had chemo and I thought ‘well, OK I am having this and it’s gonna make me better’, this time I am having it and it’s going to keep me alive a bit longer, but it’s not gonna make me better, so there’s a different kind of mindset.

‘“Chronic” resides between the category of cured and the category of terminal’, wrote Susan Gubar (2014). In the metastatic phase, therapies can no longer cure the disease; they can only push the terminal stage and death further away and keep a patient alive ‘a bit longer’, but exactly how longer? While the interviews with the medical professionals show the concept of chronicity as irregular and blurred as a nebula – and attempts to define it often indirect – the interviews with the patients show another side to the uncertainty. The patients and their illness experiences are, so to say, inside the nebula. They try to orient themselves and to manage their condition, but the information the medical professionals can give them is not enough to know what the development of their illness will be. Kathy’s experience shows the difficulty of navigating the uncertainty of survival time. She was in her fifties when she was diagnosed with metastatic breast cancer, with metastases already spread in several organs. Discussing the moment of the diagnosis, she said, ‘they told me the cancer was not curable but treatable, and I remember asking how long do I have, thinking that maybe I only had a few months, and they said "well, we hope you have a few years"’. This information seemed vague as well as in contradiction with other suggestions Kathy had received from the professionals:

I had only been told a week earlier that I had… out of the blue, that I was going to die of cancer, and she [the secondary breast cancer nurse] started talking to me about palliative care, which in my mind means ‘you are dying, you are dying, you are in a hospice’ […] And she also said I needed to get in touch with a hospice… […] what she meant was that I should start to become familiar with the hospice […]. I wasn’t so unwell [that] I needed to stay there, but I should start to become used to the idea of the hospice […]. It took a whole year longer to come to terms with what I was told.

Like Kathy, several other patients had also been told to contact a hospice; according to Dr Nadia, this is commonly suggested to patients with MBC because a palliative care team can help with pain management (cf. Kabel 2013 on the outpatient use of hospice services). However, the idea that a patient might live a few years seems to contradict the suggestion of contacting a hospice, encapsulating the unstable and nebulous temporality that MBC introduces to a patient’s life. For many women, some of the most difficult challenges are making sense of this new uncertainty and trying to understand how to situate themselves. Rather than a continuum between chronicity and terminality, they can experience a contradiction in which they are simultaneously chronic and terminal. Several patients mobilised the argument used by medical professionals, affirming that an internal variety exists within the category of MBC. According to Mary, a woman in her fifties whose cancer had spread to the bones,

A surgeon […] actually helped me put [my diagnosis] a bit more in context, i.e. it’s stage four, which is bad, but it depends where you are in stage four. There’s a range of stage four, and I’m at the moment not there, I will be, one day, but at the moment I’m at the… earlier stages of stage four. […] ‘Cause it is not black and white, is it? […] It comes in different shapes and sizes, ultimately […] you die, but it can be a longer process for some than for others.

Several patients reiterated that, although MBC is not curable, several therapies are now available to help them survive longer. Although the idea of MBC as a chronic condition was mentioned in some cases, this was often in passing and with some doubts. For example, according to Nancy, ‘We [patients with MBC] are living with… some people would call it a chronic illness, but it never lasts a life, a limiting illness’. When patients used the term chronic to describe their condition, it was usually for lack of a better definition. Among the women I met, only Letitia, a patient in her early seventies, discussed MBC’s potential definition as chronic in detail:

But this question of cancer itself being seen as a chronic condition, and having a lot in common with other diseases… rather than being something completely special, and on its own… Particularly the living with uncertainty bits, not knowing how long you’ve got, not knowing how long you will be fit and well… Living with something that is… actually doesn’t ever go away, even if you haven’t got secondary disease, you might have or you might be worrying about it, not telling anybody, which a lot of people, a lot of people do. Or a lot of people get overwhelmed by the anxiety, yeah. I think we don’t know the full scale of it yet.

Letitia was the only patient who had been living with MBC for more than 10 years. Hers was one of the successful cases that the doctors refer to when they say that ‘for some patients’, MBC can be a chronic disease. Therefore, this might have been a reason chronicity was relevant for Letitia. The concept of chronicity enabled Letitia to link her experience with the experience of people with other diseases, and not necessarily with cancer. However, she also highlighted another burden linked to the experience of chronicity, suggesting that its uncertain character weighs on the lives of patients, having an impact not only on the body but also on the mind. Organising one’s life around a disease that requires constant therapies produces continuous anxiety; according to Letitia, that anxiety is often underestimated.

## The unstable success in MBC

In these pages, I have discussed the different positions that medical literature, doctors and patients take on chronicity and how the concept of the nebula is helpful for understanding chronicity in the case of MBC.

Considering MBC as chronic can be seen as a way to locate MBC within a new biomedical paradigm; such a movement can be understood by taking into account the broader history of success and failures within cancer research. I have shown how medical literature’s presentation of MBC as chronic can constitute a way of including it in the optimistic rhetoric surrounding breast cancer. Most patients reject the notion of chronicity, sometimes perceiving it as part of a rhetoric that dismisses their suffering. Many of the medical professionals interviewed also voiced doubts.

For medical professionals, the attempt is to account for therapeutic improvements that have increased the survival times for some patients but that ultimately do not prevent almost all patients with MBC dying from it. The concept of chronicity is used tentatively by them to identify certain defining lines – or gravitational forces. MBC could be described as chronic for subtypes of cancer with longer median survival times, and it could be useful to consider MBC chronic to give more attention to patients’ quality of life, but at the same time MBC could not be chronic because it does not follow the development of typical chronic diseases (e.g. diabetes) and because it cannot be treated by non-specialists. While the main use of the concept of chronicity seems therefore to reinscribe MBC in an optimistic discourse about breast cancer, further uses are linked to discussing how the condition should be managed and to make it intelligible when compared to other diseases. The patients were inside the nebula: they tried to manage their condition following the information received from medical professionals, but they could not anticipate what would happen to them personally. For some patients, chronicity was part of the economy of hope (Delvecchio Good et al. 1990) or enabled connection with patients with other illnesses. However, for most patients, presenting MBC as chronic diminished their experience of illness.

With the improvements in survival times in MBC, there are attempts to extend chronicity to the condition, but doing so has stretched the concept of chronicity itself much closer to the moment of death. In this context, the keyword is ‘treatable’, indicating that MBC cannot be cured but can be controlled for variable periods using an increasingly broad set of treatment options. For many patients, the extension of survival time depends on debilitating treatments that are often received within the hospital.

The analysis here presented is a critique of the concept of chronicity from an anthropological perspective. Comparing the concept with a nebula, an unstable compound shaped by gravitational forces, is useful to interrogate the clarity and objectivity often ascribed to biomedical terminology. The analysis conducted shows not only the instability of ‘chronicity’ in relation to the concepts of acute and terminal, but also how the concept of chronicity itself is characterised by the co-presence of several lines of interpretation that do not coalesce into a shared definition. Despite this, chronicity is a widespread and powerful idea that in the context of breast cancer in particular can be employed to normalise the metastatic phase. The word ‘chronic’ was used by some of the patients I interviewed with hesitation, and not because it was considered appropriate or precise, but because of a lack of a better or more appropriate concept. This shows how chronicity is not a neutral and purely descriptive term but a charged concept that shapes expectations regarding illness and can limit the recognition of individual experiences.
